# A Case of Severe Acute Kidney Injury Exacerbated by Canagliflozin in a Patient with Type 2 Diabetes

**DOI:** 10.1155/2019/8639629

**Published:** 2019-06-04

**Authors:** Kimya Hassani-Ardakania, Mark L. Lipman, Denny Laporta, Oriana Hoi Yun Yu

**Affiliations:** ^1^Department of Medicine, Jewish General Hospital, McGill University, Montreal, H3T 1E2, Canada; ^2^Division of Nephrology, Department of Medicine, Jewish General Hospital, Montreal, H3T 1E2, Canada; ^3^Division of Adult Critical Care, Department of Medicine, Jewish General Hospital, Montreal, H3T 1E2, Canada; ^4^Centre for Clinical Epidemiology, Lady Davis Institute, Jewish General Hospital, Montreal, H3T 1E2, Canada; ^5^Division of Endocrinology, Department of Medicine, Jewish General Hospital, Montreal, H3T 1E2, Canada

## Abstract

**Background:**

Sodium glucose cotransport (SGLT)-2 inhibitors are the newest class of antihyperglycemic agents used as second- or third-line treatment in the management of type 2 diabetes. Although the use of SGLT-2 inhibitors has not been shown to cause nephrotoxicity, there have been case reports of SGLT-2 inhibitor use being associated with acute kidney injury.

**Case Presentation:**

A 72-year-old woman with a history of type 2 diabetes and no known chronic renal insufficiency presented to the emergency room with a 3-day history of nausea, vomiting, and increased somnolence. She was found to have potassium level of 7.4 (normal: 3.5-5.5) mmol/L and a markedly elevated creatinine level at 1154 (normal: 45-95) *μ*mol/L. Imaging of the abdomen and pelvis did not reveal any findings of obstruction. Urine microscopy showed many granular casts. In the absence of other causes for her clinical presentation, the patient was diagnosed with acute kidney injury secondary to ischemic acute tubular necrosis, with canagliflozin use likely an important contributing factor.

**Conclusions:**

Physicians should inform patients to stop the use of SGLT-2 inhibitors when patients are unable to maintain hydration or during acute illness. Use of SGLT-2 inhibitors in managing type 2 diabetes should be done with caution among more vulnerable populations, including individuals with cognitive impairment and the elderly.

## 1. Introduction

Sodium glucose cotransporter-2 (SGLT-2) inhibitors, including canagliflozin, empagliflozin, and dapagliflozin, are the newest antihyperglycemic agents approved for treatment of type 2 diabetes. The EMPA-REG trial [1] and a subsequent post hoc analysis of renal outcomes among patients with chronic renal insufficiency reported that empagliflozin reduced cardiovascular outcomes and slowed progression of kidney disease, respectively [2]. Similarly, the CANVAS trial demonstrated that patients treated with canagliflozin had a lower risk of cardiovascular events and renal outcomes [3]. The DECLARE-TIMI trial showed a decrease in the risk of acute kidney injury (AKI) associated with the use of dapagliflozin treatment [4].

More recently, the CREDENCE trial [5] found a significantly decreased risk of renal outcomes which were a composite of end stage renal disease, a doubling of creatinine levels, or death from cardiovascular or renal causes associated with the use of low dose canagliflozin (100mg daily) compared to placebo among patients with diabetes and albuminuric chronic kidney disease (with an estimated glomerular filtration rate of 30 to <90 ml per minute per 1.73m^2^ of body surface area and urinary albumin [milligrams]-to-creatinine [grams] ratio of >300 to 5000). While these large trials have demonstrated positive impact of SGLT-2 inhibitors on renal function, findings from clinical trials are not necessarily reflective of the realities of clinical practice. Indeed, several case reports have linked acute renal injury to use of SGLT-2 inhibitors including one recent report of acute renal injury with biopsy proven acute tubular necrosis (ATN) associated with the use of dapagliflozin [6]. As a result, the United States Food and Drug Administration (FDA) strengthened the warning on the risk of AKI associated with canagliflozin and dapagliflozin following assessment of these cases [7].

The following case illustrates an example of AKI that was exacerbated or potentially caused by the use of SGLT-2 inhibitors in a patient that was unable to maintain adequate hydration during a viral illness. This case emphasizes the importance of physicians to inform patients to stop the use of SGLT-2 inhibitors during acute illness.

## 2. Case Presentation

A 72-year-old female was admitted to the intensive care unit for AKI and severe shock. Her medical history included type 2 diabetes mellitus, Alzheimer's disease, hypertension, dyslipidemia, gastroesophageal reflux disease, and obstructive sleep apnea. The patient had no history of underlying chronic kidney disease.

During the three-day period before admission to the hospital, the patient was feeling unwell and increasingly somnolent, had significantly decreased oral intake, and was vomiting. She denied any fever, night sweats, or sick contacts. There was no history of diarrhea. Her medications included valsartan, metoprolol, rosuvastatin, aspirin, canagliflozin, sitagliptin, metformin, insulin degludec and aspart, donepezil, citalopram, gabapentin, and pantoprazole. Canagliflozin 300mg prescribed once daily was initiated approximately 18 months prior to presentation and was added to the antihyperglycemic agents that are listed. Otherwise, her medications were not changed during the 18 months prior to her presentation to the emergency room. She was not using herbal products or any other over-the-counter medications and did not ingest alcohol.

At presentation, the patient was somnolent, responding only to painful stimuli. Vital signs at presentation were the following: blood pressure 97/36 mmHg, heart rate 76 beats/min, respiratory rate 28 breaths/min, temperature 37.2°C, and SaO2 97% on nasal prongs. Physical examination was otherwise unremarkable. A Foley catheter was inserted which revealed minimal urine output. A point-of-care venous blood gas showed the following results: pH 7.00 (normal 7.35-7.45), pCO2 29 (normal 37-43 mmHg), bicarbonate 7 (normal 22-26 mmol/L), lactate 11.9 (normal 0.5-2.5 mmol/L), sodium 122 (normal 134-144 mmol/L), potassium 7.4 (normal: 3.5-5.5 mmol/L), and anion gap 48 mmol/L. There was an absence of ketones in the urinary dipstick. Laboratory evaluation revealed markedly elevated creatinine level at 1154 (normal: 45-95 *μ*mol/L). A complete blood count was unremarkable except for an elevated white blood cell count with a result of 24.5 x 10^9^/L (normal: 4-11x10^9^/L). Blood cultures did not result in any growth obtained at the time of presentation. A CT scan of the abdomen and pelvis did not show any evidence of hydronephrosis or hydroureter. Urine microscopy showed many granular casts. In the absence of other causes for her clinical presentation, the patient was diagnosed with AKI secondary to ischemic ATN, with canagliflozin use likely an important contributing factor in the presence of a possible viral infection that led to the nausea and vomiting.

**Figure 1:**
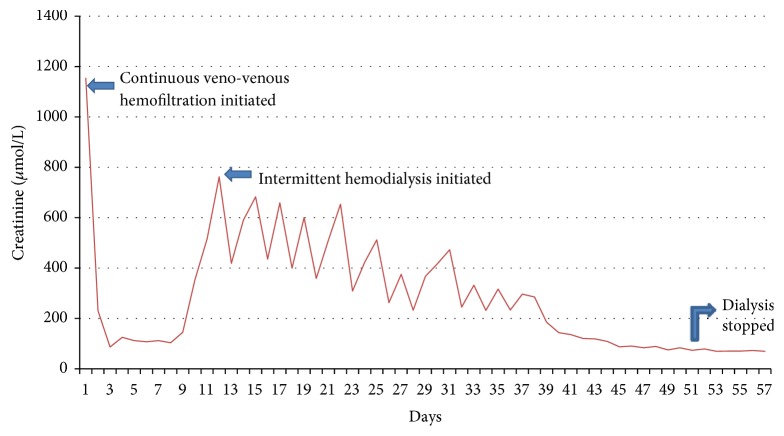
Graph showing creatinine levels from time of presentation until resolution of acute kidney injury.

She was immediately started on renal replacement therapy. She initially received continuous veno-venous hemofiltration (CVVH) and was subsequently converted to intermittent hemodialysis (IHD) when she became hemodynamically stable. The patient was anuric when the CVVH was initiated; however during the third week of her admission, the patient was noted to have a small quantity of urine output. Her renal function recovered gradually after 8 weeks of IHD (Figure 1).

## 3. Discussion

SGLT-2 inhibitors can cause kidney function decline through different mechanisms including volume depletion due to excessive diuresis, decrease in transglomerular pressure, and renal medullary hypoxic injury caused by increased distal tubular transport [9]. These effects become further amplified in the setting of volume depletion and underlying renal impairment [10]. Therefore, SGLT-2 inhibitors could induce AKI from prerenal failure caused by increased diuresis and ATN has also been described to occur secondary to renal hypoxic injury induced by the use of these agents [9, 11].

The FDA has raised concerns for AKI associated with SGLT-2 inhibitor use. More recently, an analysis of the FDA adverse event report system database found that patients with type 2 diabetes on SGLT-2 inhibitors were at a significantly greater risk of AKI compared to those who were not treated with SGLT-2 inhibitors [10]. Among the SGLT-2 inhibitors, canagliflozin was associated with the highest proportion of renal failure. Interestingly, cases of renal failure with SGLT-2 inhibitors were four times more likely to report concomitant use of angiotensin converting enzyme (ACE) inhibitors, angiotensin II receptor blockers (ARBs), and diuretics, compared to cases with SGLT-2 inhibitors reporting other adverse events [10].

A meta-analysis of 55 randomized clinical trials, performed in 2013, demonstrated that canagliflozin and dapagliflozin use was associated with an increased risk of AKI compared to use of non-SGLT-2 inhibitor antihyperglycemic agents [12]. The findings from this systematic review and meta-analysis showed that patients with moderate renal impairment had a higher incidence of renal adverse events when treated with canagliflozin or dapagliflozin. Furthermore, patients with normal to mild renal impairment had a higher incidence of renal adverse events if they were treated with the higher dose of canagliflozin (i.e., 300mg daily). However, three subsequent large randomized control trials studying cardiovascular outcomes associated with the use of empagliflozin [1], canagliflozin [3], and dapagliflozin [4] found no increased risk of AKI among individuals treated with SGLT2 inhibitors compared to placebo. In fact, the DECLARE-TIMI58 trial, which studied dapagliflozin, found a decreased risk of renal events among patients treated with dapagliflozin compared to patients treated with placebo (hazard ratio [HR]: 0.69; 95% confidence interval [CI]: 0.55–0.87) [4]. More recently, the CREDENCE trial studied adverse renal outcomes among patients with type 2 diabetes and albuminuric chronic kidney disease and found that low dose canagliflozin (100mg daily) was associated with lower risk of adverse renal outcomes defined by a composite of end stage renal disease, a doubling of creatinine levels, or death from cardiovascular or renal causes (HR: 0.66; 95% CI: 0.53-0.81) [5]. These findings are further supported by real-world data from the Mount Sinai chronic kidney disease registry and the Geisinger Health System cohort that assessed the risk of AKI associated with the use of SGLT-2 inhibitors compared to use of non-SGLT-2 inhibitor antihyperglycemic agents using propensity-score matched analyses [13]. However, the authors acknowledge that SGLT-2 inhibitor use may be associated with AKI among high-risk individuals, which were not studied in this observational study cohort.

In this case report, we believe that canagliflozin played an important causative role in our patient's AKI in addition to her acute viral illness, poor oral intake, and concomitant use of valsartan. Unfortunately, it is difficult to determine whether the AKI was caused by a prerenal mechanism as urinary sodium levels were not obtained at presentation. However, the microscopic urinary analysis showed many granular casts, which suggested that the AKI was caused by ATN. To our knowledge, this is the second case report of AKI secondary to ATN that was associated with the use of SGLT-2 inhibitor [6].

**Figure 2:**
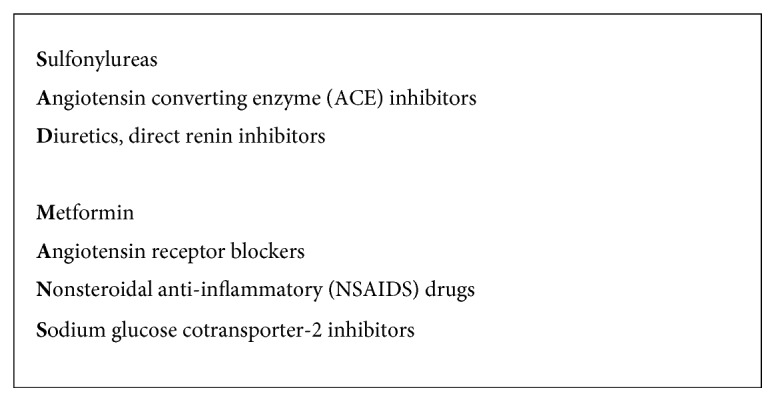
Sick day medication list. Figure adapted from Diabetes Canada [8].

In conclusion, this case report illustrates an example of canagliflozin use contributing to AKI in the event of an acute illness. Given the efficacy of SGLT-2 inhibitors and their cardiovascular benefits, their usage in patients with type 2 diabetes is well justified. However, given that there are plausible mechanisms linking SGLT-2 inhibitors to AKI, especially when having concomitant use of ACE inhibitors, ARBs, diuretics, or other nephrotoxic medications such as nonsteroidal anti-inflammatory drugs, amphotericin, and radiocontrast media [9], it is important to consider this adverse effect when prescribing SGLT-2 inhibitors. Moreover, usage of SGLT-2 inhibitors should be done with caution in elderly patients [14] and in patients with cognitive impairment as they may not be able to maintain hydration or remember to stop SGLT-2 inhibitors in the event of acute illness. Finally, given that SGLT-2 inhibitor use can exacerbate volume depletion via its diuretic effects, resulting in renal function decline during illness, guidelines from Diabetes Canada have integrated SGLT-2 inhibitors as one of the class of medications that should be stopped during acute illness (Figure 2) [8]. As SGLT-2 inhibitors are becoming more frequently prescribed due to the metabolic and cardiovascular benefits in the treatment of type 2 diabetes, it is important for clinicians to inform their patients of the potential side effects to decrease the complications associated with the use of these agents.

## Abbreviations

SGLT-2: Sodium glucose cotransporter-2
AKI: Acute kidney injury
ATN: Acute tubular necrosis
FDA: Food and Drug Administration
CVVH: Continuous veno-venous hemofiltration
IHD: Intermittent hemodialysis
ACE: Angiotensin converting enzyme
ARB: Angiotensin II receptor blockers.
