# Corrigendum to “A Case of Severe Acute Kidney Injury Exacerbated by Canagliflozin in a Patient with Type 2 Diabetes”

**DOI:** 10.1155/2021/7320217

**Published:** 2021-02-04

**Authors:** Kimya Hassani-Ardakani, Mark L. Lipman, Denny Laporta, Oriana Hoi Yun Yu

**Affiliations:** ^1^Department of Medicine, Jewish General Hospital, McGill University, Montreal H3T 1E2, Canada; ^2^Division of Nephrology, Department of Medicine, Jewish General Hospital, Montreal H3T 1E2, Canada; ^3^Division of Adult Critical Care, Department of Medicine, Jewish General Hospital, Montreal H3T 1E2, Canada; ^4^Centre for Clinical Epidemiology, Lady Davis Institute, Jewish General Hospital, Montreal H3T 1E2, Canada; ^5^Division of Endocrinology, Department of Medicine, Jewish General Hospital, Montreal H3T 1E2, Canada

In the article titled “A Case of Severe Acute Kidney Injury Exacerbated by Canagliflozin in a Patient with Type 2 Diabetes” [1], there was a spelling error in the author Kimya Hassani-Ardakani's name in the author list, where Kimya Hassani-Ardakania should have read Kimya Hassani-Ardakani. This is corrected as shown above.
